# Private sector drug shops frequently dispense parenteral anti-malarials in a rural region of Western Uganda

**DOI:** 10.1186/s12936-018-2454-7

**Published:** 2018-08-22

**Authors:** Lawrence T. Wang, Robert Bwambale, Corinna Keeler, Raquel Reyes, Rabbison Muhindo, Michael Matte, Moses Ntaro, Edgar Mulogo, Radhika Sundararajan, Ross M. Boyce

**Affiliations:** 1 School of Medicine, University of California San Diego, 9500 Gilman Drive, La Jolla, CA 92093 USA; 2 Bugoye Level III Health Centre, Uganda Ministry of Health, Bugoye, Kasese District, Western Region Uganda; 3 Department of Geography, University of North Carolina at Chapel Hill, Campus Box 3220, Chapel Hill, NC 27599 USA; 4 Division of General Medicine & Clinical Epidemiology, University of North Carolina at Chapel Hill, 5039 Old Clinic Building, CB 7110, Chapel Hill, 27599 USA; 5 Department of Community Health, Mbarara University of Science & Technology, P.O. Box 1410, Mbarara, Uganda; 6 Department of Emergency Medicine, Weill Cornell Medical College, 525 East 68th Street, New York, NY 10065 USA; 7 Division of Infectious Diseases, University of North Carolina at Chapel Hill, 130 Mason Farm Road, Chapel Hill, NC 27599 USA

**Keywords:** Malaria, Health-seeking behaviors, Private sector, Anti-malarials, Parenteral drugs

## Abstract

**Background:**

Malaria is a leading cause of paediatric morbidity and mortality in Uganda. More than half of febrile children in rural areas initially seek care at private clinics and drug shops. These shops are generally unregulated and the quality of clinical care is variable, with the potential for misdiagnosis and the development of drug resistance. There is thus an urgent need to identify rural drug shops and coordinate their malaria treatment efforts with those of the public sector. The objective of the study was to identify all drug shops in the Bugoye sub-county of Western Uganda and assess their anti-malarial dispensing practices.

**Methods:**

This study is a cross-sectional survey of drug shops in a rural sub-county of Western Uganda. In the first phase, shop locations, licensing and shopkeeper’s qualifications, and supply and pricing of anti-malarials were characterized. In the second phase, the proportion of anti-malarials dispensed by private drug shops was compared to public health facilities.

**Results:**

A total of 48 drug shops were identified. Only one drug shop (1 of 48, 2%) was licensed with the sub-county’s records office. The drug shops stocked a variety of anti-malarials, including first-line therapies and less effective agents (e.g., sulfadoxine/pyrimethamine). Almost all drug shops (45 of 48, 94%) provided parenteral anti-malarials. Of the 3900 individuals who received anti-malarials during the study, 2080 (53.3%) purchased anti-malarials through the private sector compared to 1820 (46.7%) who obtained anti-malarials through the public sector. Drug shops were the primary source of parenteral anti-malarials. Inadequate dosing of anti-malarials was more common in drug shops.

**Conclusions:**

Drug shops are major sources of parenteral anti-malarials, which should be reserved for cases of severe malaria. Strengthening malaria case management and incorporating drug shops in future interventions is necessary to optimize malaria control efforts in the sub-county, and in similarly endemic regions.

**Electronic supplementary material:**

The online version of this article (10.1186/s12936-018-2454-7) contains supplementary material, which is available to authorized users.

## Background

Approximately 30–50% of outpatient visits to public healthcare facilities in Uganda are related to malaria, which remains a leading cause of paediatric morbidity and mortality [1, 2]. However, the private sector is also an important source of anti-malarials in rural Uganda, where geographic factors such as distance to health centres and weak transportation networks can limit access to medical care [2–6]. More than half of febrile children in rural areas initially seek care at private drug shops, which are often unregistered, unregulated, and operated by individuals with limited formal clinical training [7–13]. A census of drug shops in rural Uganda found that 77% were unlicensed [8] and a national survey that tracked anti-malarial dispensation in Uganda’s private sector found that 20.0% of drug shops sold parenteral anti-malarials, a practice that is contrary to the policies of Uganda’s Ministry of Health [14].

Drug shops have also been found to market expired and/or ineffective anti-malarials despite the availability of efficacious first-line therapies, a practice that may result in adverse clinical outcomes [15–18]. One study found that licensed rural drug shops provided appropriate malaria treatments to only 10% of febrile children [7], and another study demonstrated that initially seeking care at a drug shop was a significant risk factor for severe malaria in Ugandan children [19]. These factors contribute to the highly variable quality of malaria case management in drug shops [7, 17, 19–22].

Drug shops do have several advantages compared to public facilities, especially in a rural context. They are generally more accessible due to their proximity to village population centres, shorter waiting times, and more flexible hours of operation. They are also less prone to drug stock-outs [23–27]. When provided with training in basic case management practices, drug shops increased patient access to appropriate care [10, 28, 29]. Thus, there is an urgent need to identify rural drug shops, evaluate current practices, and coordinate malaria treatment efforts with national guidelines and priorities.

This study was a cross-sectional survey examining the characteristics of drug shops in Bugoye sub-county, a rural area of highland western Uganda whose private sector has never been assessed. The objective of the study was to identify all drug shops in the sub-county and assess their anti-malarial dispensing practices in comparison to neighboring public health facilities.

## Methods

### Study setting

**Fig. 1:**
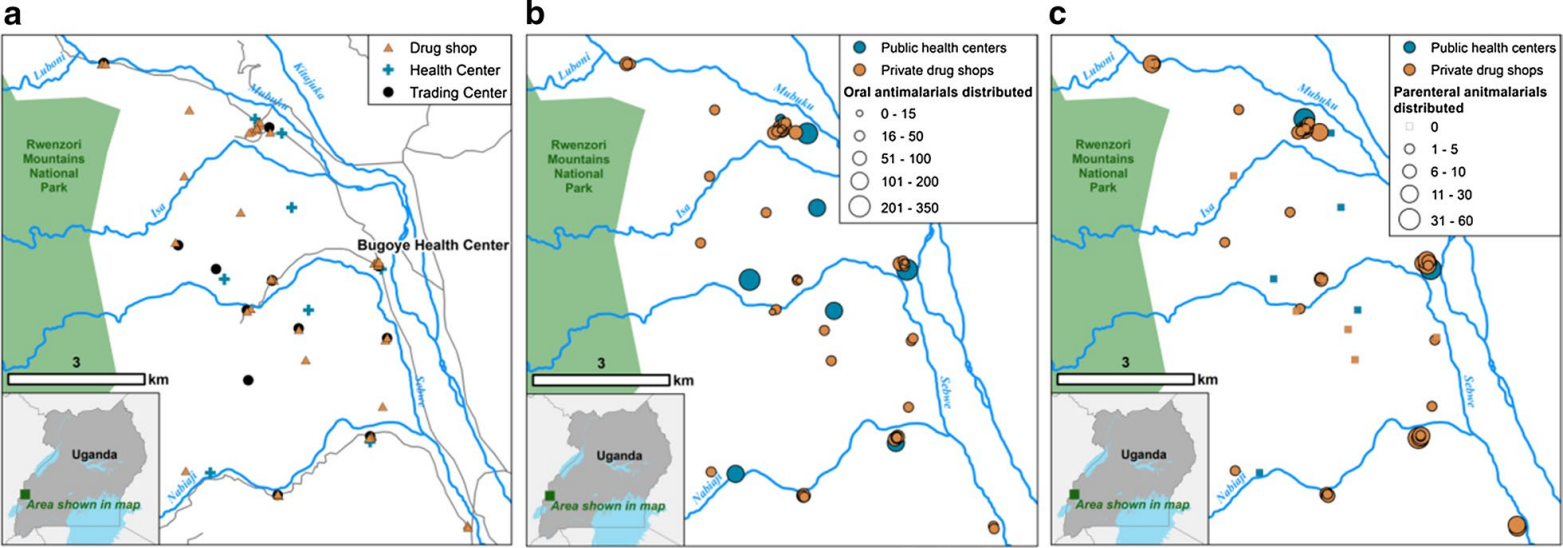
Map depicting the locations of public sector health centres and private sector drug shops in Bugoye sub-county (**a**), with number of patients receiving oral anti-malarials during the study period (**b**), and number of patients receiving parenteral anti-malarials during the study period (**c**)

The study was conducted in Bugoye sub-county, Kasese District, Western Uganda (Fig. 1). The highland terrain, with elevations ranging from 1000 to 1800 m, is home to approximately 35,000 residents [30, 31]. Bugoye’s climate allows for year-round malaria transmission interspersed with semi-annual transmission peaks after the rainy seasons [30]. Recent surveys suggest that malaria transmission in the region is declining, evidenced by a parasite prevalence of 48.4% in 2009 compared to 17.6% in 2014 [2, 32]. Bugoye sub-county’s health system is divided into public and private health facilities.

#### Public health facilities

The sub-county’s primary public health facility is the Bugoye Level III health centre (BHC), which houses an inpatient ward where parenteral medications are administered, an outpatient ward where oral medications are dispensed, and a laboratory that conducts point-of-care diagnostics including malaria rapid diagnostic tests (RDTs) and light microscopy. There are Level II Health Centres in each of the six parishes that offer basic outpatient services. There are two community health workers termed village health teams (VHTs) in each of the sub-county’s 36 villages that are trained in integrated community case management (iCCM), perform RDTs, and dispense oral medications to children < 5 years of age suffering from uncomplicated pneumonia, diarrhoea, and malaria [33]. There is one public–private Level III Health Centre operated jointly by the Ugandan Ministry of Health and the Rwenzori Mountaineering Services (RMS), which houses a public outpatient ward and a private inpatient ward that provide services comparable to BHC. Remote villages were defined as those at least 3 km from BHC.

#### Private health facilities

To our knowledge, drug shops (i.e., privately owned small businesses that sell pharmaceutical products) comprise the entirety of the sub-county’s private health facilities.

### Study design

This study was a two-phase, cross-sectional survey of drug shops in Bugoye sub-county. In the first phase, shop locations, self-reported licensing status and shopkeeper’s qualifications, and supply and pricing of anti-malarials were recorded. In the second phase, the number of anti-malarials dispensed by private drug shops was compared to public health facilities.

#### Phase 1: initial survey of drug shops

The locations of shops in the sub-county were identified with the assistance of local leaders and VHTs. Study staff visited all villages at least twice to ensure that no shops were missed. At the initial visit, the following information was collected on a questionnaire (Additional file 1): the village and shop names, GIS coordinates, self-reported licensing status, and shopkeeper’s credentials. The following information was recorded for each anti-malarial available at a shop: the drug’s trade name, manufacturer’s country of origin, formulation and route of administration, price for an adult and paediatric course (in Ugandan shillings), expiration date, and the shop’s current drug supply. Study staff also determined the availability and cost of malaria RDTs and asked about the shop’s primary resupply source. The names of shops that claimed to be licensed were compared to a list of licensed drug shops maintained by BHC.

#### Phase 2: tracking anti-malarial dispensation in drug shops and health centres

After completing the initial visit questionnaire, a form was provided to each shopkeeper (Additional file 2) along with instructions to record anti-malarial sales for 1 month beginning on 1 August 2017. For every anti-malarial sold, shopkeepers recorded the following information: date of sale, the purchaser’s gender, age, and village of residence, the drug name, units dispensed (recorded in tablets/bottles/ampules), and the remaining stock. Shopkeepers were instructed to return the questionnaire to BHC after 31 August 2017.

Over the same period, the following information on anti-malarial dispensation was extracted from the clinical registers of all public health facilities in the sub-county: date of encounter, patient’s gender, age, village of residence, and type and dose of anti-malarial dispensed. Records of children < 5 years of age seen by VHTs conducting integrated management of childhood illness (IMCI) were collated separately by BHC staff and shared with study staff. Anti-malarial tracking forms were not provided to public facilities as records were abstracted from existing registers.

#### Anti-malarial drug courses

The definition of a course of anti-malarials was based on WHO guidelines [34]. For example, a course of oral artemether/lumefantrine (AL) for an adult > 35 kg consists of 24 tablets (each containing 20 mg artemether and 120 mg lumefantrine), with 4 tablets as a single initial dose, followed by 4 tablets after 8 h, then 4 tablets twice a day (AM and PM) for the following 2 days. For an adult > 35 kg, a course of parenteral artesunate consists of six 60 mg ampules reconstituted in 5% sodium bicarbonate and diluted in 5% (w/v) glucose. A loading dose of 2 mg/kg is followed by 1 mg/kg after 4 and 24 h; subsequently, a dose of 1 mg/kg is given daily until the patient can tolerate oral therapy [34].

### Statistical analysis

Data were entered into Microsoft Excel, version 15.33 (Microsoft, Redmond, WA) and analyzed with Stata, version 15.1 (Stata Corp., College Station, TX). A map of all health facilities was created using ArcGIS, version 10.4.1 (ESRI, Redlands, CA). The level of owner credentialing and self-reported licensing status with the sub-county’s records office were summarized, along with the availability, formulation, manufacturing origin, and median cost of each anti-malarial.

We employed common statistical tests to (1) compare patient populations receiving anti-malarials between the public and private sector, (2) explore the accuracy of AL dosing between the public and private sector, and (3) assess for significant differences in the rate of parenteral anti-malarial administration across the private sector. The demographic characteristics of patients presenting to private versus public health facilities were compared using Wilcoxon Rank Sum test for continuous variables (i.e., age) and Pearson’s Chi squared test for categorical variables.

The frequency of inadequate AL dose administration was explored in two groups: adults (defined as individuals ≥ 21 years of age with a weight assumed to be > 35 kg) and children (≤ 5 years of age with a weight assumed to be < 15 kg). AL was selected as a proxy for inadequate dosing because it is both the first-line therapy for uncomplicated malaria and the most commonly prescribed anti-malarial in the sub-county. Inadequate dosing was defined as the dispensation of < 6 AL tablets for children ≤ 5 years of age and < 24 tablets for adults ≥ 21 years of age. The proportion of inadequate dosing was compared between private and public facilities using Pearson’s Chi squared test.

Differences in the relative odds of parenteral anti-malarial administration across drug shops were examined using Poisson regression models. The outcome of interest was the route of administration (i.e., parenteral vs. oral) and the primary explanatory variable was individual drug shops, with BHC serving as the reference. Models were adjusted for age and reported as odds ratios (OR). For all statistical analyses, a resulting p-value of ≤ 0.05 was considered significant.

## Results

### Phase 1: initial survey of drug shops

#### Licensing and shopkeeper’s qualifications

A total of 48 drug shops were identified (Fig. 1a) and all shopkeepers consented to participate in the study. Only one shop (1 of 48, 2.1%) was licensed with the sub-county’s records office. Some shops (16 of 48, 33%) had more than one shopkeeper; most (48 of 67, 72%) identified themselves as nursing assistants, which in Uganda qualifies one to dispense medications under supervision.

#### Supply and pricing of anti-malarials

The drug shops stocked a variety of anti-malarials (Table 1). Nearly all shops (47 of 48, 98.0%) stocked AL at a median cost of $1.11 (IQR 1.11–1.28) for an adult course of 24 tablets. Quinine was available in tablet (43 of 48, 90%), syrup (48 of 48, 100%), and parenteral forms (45 of 48, 94%). Older agents like sulfadoxine/pyrimethamine (SP) that are no longer considered effective for the treatment of uncomplicated malaria [35] were also available in drug shop (42 of 48, 88.0%). In general, parenteral anti-malarials were more expensive, with an adult course of 6 ampules of parenteral artesunate (median cost $13.33, IQR 13.33–13.33) being the most expensive treatment. None of the anti-malarials examined on the initial visit were expired. All shops reported restocking supplies from pharmacies in Kasese Town, approximately 20 km to the south. All shops offered malaria RDTs for a median cost of $0.56 (IQR $0.56–$0.83).

**Table 1:** Anti-malarial drugs available in drug shops in Bugoye sub-county

Drug name	Formulation	Adult courses^a^	Cost^b^ (median, IQR)	Drug shops (n, %)
Oral formulations				
Amodiaquine	Tablet	9.5 (6 tablets)	2.08 (1.46–2.78)	6/48 (13)
Artemether	Tablet	0 (12 tablets)	1.81 (1.32–2.78)	2/48 (4)
Artemether/lumefantrine	Tablet	316.8 (24 tablets)	1.11 (1.11–1.28)	47/48 (98)
Chloroquine	Tablet	6.1 (10 tablets)	1.11 (0.83–1.11)	4/48 (8)
Dihydroartemisinin/piperaquine	Tablet	15.6 (9 tablets)	4.17 (4.17–6.94)	7/48 (15)
Quinine	Syrup	99 (1 bottle)	1.11 (1.11–1.39)	48/48 (100)
Quinine	Tablet	31.7 (30 tablets)	2.50 (2.08–2.50)	43/48 (90)
Sulfadoxine/pyrimethamine	Tablet	243.8 (3 tablets)	0.42 (0.42–0.54)	42/48 (88)
Parenteral formulations				
Artemether	Parenteral	6.5 (6 ampules)	4.58 (3.13–8.33)	9/48 (19)
Artesunate	Parenteral	13.8 (6 ampules)	13.33 (13.33–13.33)	22/48 (46)
Quinine	Parenteral	60.7 (3 ampules)	8.06 (5.56–8.33)	45/48 (94)

^a^Number (and definition) of adult courses available in all drug shops

^b^Cost in US dollars of a single adult course of specified anti-malarial

### Phase 2: tracking anti-malarial dispensation in drug shops and health centres

Completed tracking forms were received from all 48 drug shops. Of the 3900 individuals who received anti-malarials from private and public facilities during the study period, 2080 (53.3%) received anti-malarials through private facilities compared to 1820 (46.7%) who received anti-malarials through public facilities. Nearly all care on the weekends (503 of 515, 97.7%, Table 2) was provided by drug shops. Health centres were the primary source of AL, while drug shops dispensed more doses of every other type of anti-malarial, including less efficacious therapies such as SP and chloroquine (Table 3). All patients who received anti-malarials from a health centre had a positive RDT.

**Table 2:** Demographic characteristics of patients in the public and private sectors

	Public health centres^a^	Drug shops^b^	*p*-value
Patients (n, %)	1531 (39.3)	2080 (53.3)	–
Female (n, %)	877 (57.3)	1063 (53.3)	0.018
Age (median, IQR)	16 (9–27)	19 (9–30)	0.004^d^
Age category (n, %) (years)			
< 5	166 (10.9)	306 (14.8)	< 0.001
5–12	366 (24.1)	320 (15.5)	
> 12	989 (65.0)	1443 (69.7)	
Villages with health centre	685 (44.8)	923 (44.7)	0.97
Remote villages^c^	295 (19.3)	486 (23.6)	0.002
Weekend	12 (0.8)	503 (25.6)	< 0.001

^a^Excludes 289 (7.4%) patients < 5 years of age treated by VHTs

^b^Includes the 45 patients seen at the private RMS Level III Health Centre

^c^Defined as villages at least 3 km from the Bugoye Level III Health Centre

^d^Probability obtained by comparison of medians using Mann–Whitney two sample test

**Table 3:** Number of patients treated in the public sector versus the private sector

Formulation	Anti-malarial	Patients treated^b^ (n, %)	
		Public sector 1820 (46.7)	Private sector 2080 (53.3)
Tablets	Amodiaquine	0	0
	Artemether	0	5 (0.2)
	Artemether/lumefantrine	1715 (94.2)	1029 (49.5)
	Chloroquine	0	3 (0.1)
	Dihydroartemisinin/piperaquine	0	19 (0.9)
	Quinine	2 (0.1)	169 (8.1)
	Sulfadoxine/pyrimethamine	96 (5.3)	341 (16.4)
Parenteral	Artemether	0	31 (1.5)
	Artesunate	47 (2.6)	73 (3.5)
	Quinine	12 (0.7)	285 (13.7)
Syrup	Quinine	0	164 (7.9)
Rectal	Artesunate	7 (0.4)^a^	0

^a^All seven individuals were children < 5 years of age treated by VHTs

^b^The aggregate number of patients treated is less than the numbers reported in the table because some patients received more than one type of anti-malarial

#### Geographic characteristics of drug shops

Most shops distributing anti-malarials were located within 1 km of a health centre (Fig. 1b, c). Individuals from villages with a health centre were equally as likely to obtain anti-malarials from a drug shop as those from villages without a health centre (AOR 1.00, 95% CI 0.88–1.15, p = 0.97, Table 2). Conversely, individuals from remote villages furthest from BHC were more likely to obtain anti-malarials from drug shops (AOR 1.28, 95% CI 1.09–1.51, p = 0.002, Table 2).

#### Patient characteristics

Individuals receiving all types of anti-malarials from drug shops were slightly older than those that received all types of anti-malarials through health centres (median age 19 vs. 16 years, p = 0.004, Table 2). One half of individuals (165 of 329, 50.2%) treated with SP in drug shops were male, suggesting that intermittent treatment of malaria in pregnancy (IPTp) was not the rationale for selecting SP.

#### Parenteral anti-malarial dispensation

**Fig. 2:**
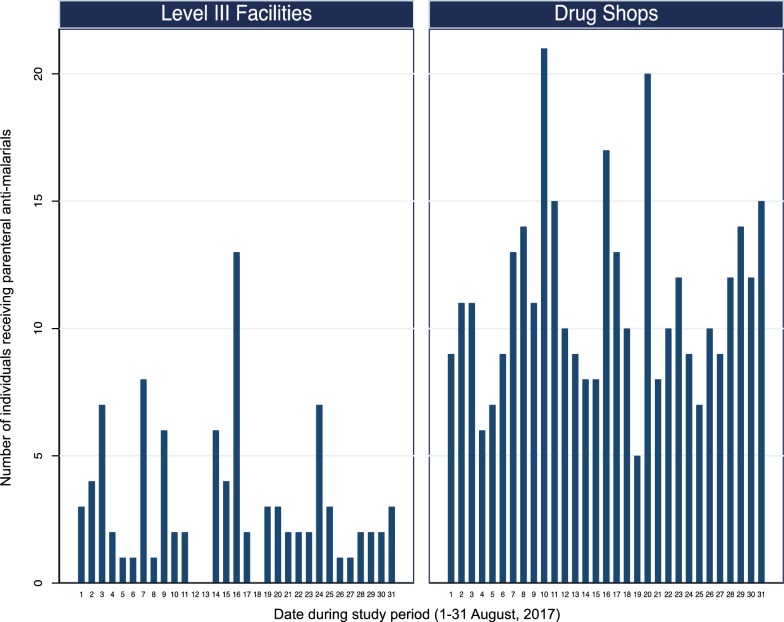
Number of individuals receiving parenteral anti-malarial therapy at the Bugoye Level III Health Centre compared to private sector drug shops during the study period (1–31 August 2017)

59 of 299 (19.7%) individuals treated for malaria at BHC received parenteral anti-malarials, consisting of either artesunate (47 of 59, 79.7%) or quinine (12 of 59, 20.3%). An additional 36 of 45 (80.0%) patients treated in the private inpatient ward of the RMS health centre received parenteral anti-malarials. All patients who received a course of parenteral anti-malarials at a Level III health centre also received a course of oral anti-malarials. Together, these two Level III facilities accounted for only 95 of the 448 (21.2%) individuals that received parenteral anti-malarials over the study period (Fig. 2).

In contrast, drug shops accounted for 353 of the 448 (78.8%) individuals that received parenteral anti-malarials over the study period (Fig. 2). The administration of parenteral anti-malarials in drug shops was common practice (Fig. 1c), with most shops (40 of 48, 83.4%) dispensing parenteral anti-malarials to at least one person over the study period. Quinine was the most frequently sold parenteral anti-malarial (251 of 353, 71.7%), followed by artesunate (71 of 353, 20.1%) and artemether (31 of 353, 8.9%). None of the patients who purchased parenteral anti-malarials from a shop purchased a concomitant course of oral anti-malarials. Five drug shops (5 of 48, 10.4%) accounted for 37.7% of individuals treated with parenteral anti-malarials in the private sector.

In the regression analysis, receiving care at drug shops was associated with significantly higher odds of parenteral anti-malarial use (AOR 5.05, 95% CI 3.80–6.72, p < 0.001) compared to BHC. At eight shops, the odds of parenteral anti-malarial sales were significantly higher (p < 0.05) or trended higher (p < 0.1) than that observed at BHC. Together, these eight shops accounted for 31.3% of individuals treated with parenteral anti-malarials in both the public and private sectors. Of these eight shops, four were in trading centres adjacent to BHC and two were near RMS.

Individuals receiving parenteral treatment in drug shops were on average 7.8 years older than those receiving treatment at BHC (22.7 vs. 15.0 years, p < 0.001). Similarly, children < 5 years of age presented less frequently to drug shops compared to BHC to receive parenteral therapy (30.8% vs. 69.2%, p < 0.001). There were no significant differences by gender.

#### Inadequate AL dosing in drug shops

Inadequate AL dosing among children was not observed in health centres (0 of 137 cases, 0.0%). However, inadequate AL dosing was relatively frequent in drug shops (11 of 94 cases, 11.7%). Inadequate AL dosing among adults was also more common at drug shops (65 of 410 cases, 15.9%) compared to health centres (8 of 526 cases, 1.5%, p < 0.001). There was no significant difference in the age of adults between private and public facilities (p = 0.80) that might explain the difference in AL dosing. Furthermore, adult women (who may weigh less than the 35-kg threshold for a 24 tablet AL course) comprised a higher, albeit non-significant, proportion of attendees at public compared to private health facilities (57.2 vs. 52.4, p = 0.14), which makes this unlikely to explain the observed differences.

## Discussion

This study demonstrates that private sector drug shops are not only significant distributors of anti-malarials in a rural area of western Uganda, but also a major source of parenteral anti-malarials. Although Uganda’s National Drug Authority does not authorize drug shops to dispense parenteral medications, a 2015 national survey found that 20.0% of drug shops sold parenteral quinine and 10.9% sold parenteral artemether. Ultimately, 8.7% of patients received non-artemisinin therapies (i.e., parenteral/tablet/syrup quinine, SP tablets, or chloroquine tablets) and 3.9% of patients received an artemisinin monotherapy (i.e., parenteral artemether) during the national survey’s data collection period [14]. The present study found that both the percentage of drug shops selling parenteral anti-malarials and the percentage of patients receiving parenteral anti-malarials were greater than previously reported. Even shops located near Level III facilities had high rates of parenteral anti-malarial sales, indicating a strong preference for seeking care in the private sector.

Several factors may account for increased utilization of drug shops in Bugoye sub-county. Long wait times at public health facilities, coupled with the convenience of seeking care in drug shops, likely led to increased acquisition of parenteral anti-malarials through the private sector. Patients may believe that parenteral anti-malarials are more efficacious than oral formulations, a perception that has been shown in other countries [36]. Furthermore, this study found that older patients were not only more likely to seek care in drug shops, but also more likely to purchase parenteral anti-malarials without an accompanying course of oral anti-malarials. On the other hand, paediatric patients were less likely to present to a shop and receive parenteral therapy. This could be because parents, concerned that their child might be suffering from severe malaria, seek formal treatment from a public facility staffed by more qualified personnel.

These results have important public health implications. In terms of patient safety, the dispensation of parenteral anti-malarials in drug shops raises concerns about safety practices, management of adverse drug effects, and possible dispensation of counterfeit drugs. In particular, parenteral quinine can have dangerous adverse effects if improperly administered. In terms of anti-malarial stewardship, all drug shops dispensed a course of parenteral anti-malarials without a concomitant course of oral anti-malarials, a practice that can lead to the development of drug resistance. Additionally, previous studies have found that initially seeking care at drug shops was a significant risk factor for severe malaria in Ugandan children, likely due to their provision of sub-optimal treatments and resultant disease progression [19, 37].

Several approaches could be taken to address these complex issues. A ban on parenteral anti-malarial dispensation in drug shops could be enforced, but may push sales further underground, where they will be even more difficult to regulate. Another option is a harm reduction approach, which could include more training for shopkeepers and more rigorous enforcement of licensing and RDT usage. The results of our analysis indicate that targeting a few high-volume drug shops could have an exponential impact on malaria case control.

This study has several methodological strengths, including its rigorous field practices identifying drug shops and its high response rate. This study also has some limitations. First, the data was drawn from clinic records in public facilities and from self-reported records in drug shops whose accuracy were not assessed. However, the rate of completeness was high and there is no reason to believe there were systemic issues in reporting. Second, study staff did not interview customers and thus can only speculate on the motivations driving their care-seeking behaviors. These behaviors can be examined in future qualitative studies. Third, this study did not track the degree of overlapping patient utilization of the public and private sector. Finally, RDT utilization in drug shops was not assessed and it is unknown whether the 2080 individuals that received anti-malarials through the private sector actually had clinical malaria. Regardless, these limitations do not diminish the striking finding that the majority of parenteral anti-malarials were dispensed through private drug shops.

## Conclusions

Private sector drug shops in Bugoye sub-county distributed more than half of all anti-malarials during the study period and were the primary source of parenteral anti-malarials. There were several risks associated with seeking care in drug shops, including higher rates of inadequate anti-malarial dosing. Further studies are needed to assess the potential risks and benefits of incorporating drug shops as stakeholders in malaria case management in Uganda.

## Additional files

**Additional file 1.** Initial visit questionnaire.

**Additional file 2.** Anti-malarial sales tracking form.
