# Supplementary material for: Private sector drug shops frequently dispense parenteral anti-malarials in a rural region of Western Uganda
Source: Malar J. 2018 Aug 22;17:305. doi: 10.1186/s12936-018-2454-7 (PMC6106765; doi:10.1186/s12936-018-2454-7)
Supplement: Supplementary file 1 — Additional file 1. Initial visit questionnaire. [file 12936_2018_2454_MOESM1_ESM.docx]

Additional File 1: Initial visit questionnaire

| **Visit Date (**Day – Month – Year): --- --- | | | | | | |
| --- | --- | --- | --- | --- | --- | --- |
| **SECTION 1 of 2: Pharmacy/Drug Shop Information** | | | | | | |
| **Shop Name:** |  | | | | | |
| **Village Name:** |  | | | | | |
| **GPS Coordinates:** |  | | | | | |
| **SECTION 2 of 2: Anti-Malarial Drugs** | | | | | | |
| **Drug Name:** | **Manufacturer/Location:** | **Formulation:** | **Stock:** | **Price:** | **Expired?** | **Malaria RDT? Cost?** |
| 🞎 Artemether (Artimisa) |  |  |  |  |  |  |
|  |  |  |  |  |  |  |
|  |  |  |  |  |  |  |
|  |  |  |  |  |  |  |
| 🞎 Artemether+Lumefantrine (Coartem) |  |  |  |  |  |  |
|  |  |  |  |  |  |  |
|  |  |  |  |  |  |  |
|  |  |  |  |  |  |  |
| 🞎 Artesunate (Plasmotrim) |  |  |  |  |  |  |
|  |  |  |  |  |  |  |
|  |  |  |  |  |  |  |
|  |  |  |  |  |  |  |
| 🞎 Artesunate+Amodiaquine (Camoquin) |  |  |  |  |  |  |
|  |  |  |  |  |  |  |
|  |  |  |  |  |  |  |
|  |  |  |  |  |  |  |
| 🞎 Chloroquine (Aralen) |  |  |  |  |  |  |
|  |  |  |  |  |  |  |
|  |  |  |  |  |  |  |
|  |  |  |  |  |  |  |
| 🞎 Dihydroartemisinin+Piperaquine (Eurartisim) |  |  |  |  |  |  |
|  |  |  |  |  |  |  |
|  |  |  |  |  |  |  |
|  |  |  |  |  |  |  |
| 🞎 Mefloquine (Lariam) |  |  |  |  |  |  |
|  |  |  |  |  |  |  |
|  |  |  |  |  |  |  |
|  |  |  |  |  |  |  |
| 🞎 Atovoquone/Proguanil (Malarone) |  |  |  |  |  |  |
|  |  |  |  |  |  |  |
|  |  |  |  |  |  |  |
|  |  |  |  |  |  |  |
| 🞎 Quinine (Qualaquin) |  |  |  |  |  |  |
|  |  |  |  |  |  |  |
|  |  |  |  |  |  |  |
|  |  |  |  |  |  |  |
| 🞎 Sulfadoxine+pyrimethamine (Fansidar) |  |  |  |  |  |  |
|  |  |  |  |  |  |  |
|  |  |  |  |  |  |  |
|  |  |  |  |  |  |  |
| 🞎 Other(s)? |  |  |  |  |  |  |
|  |  |  |  |  |  |  |
|  |  |  |  |  |  |  |
|  |  |  |  |  |  |  |
|  |  |  |  |  |  |  |
|  |  |  |  |  |  |  |
|  |  |  |  |  |  |  |
|  |  |  |  |  |  |  |

**Notes:**
