# Supplementary material for: Private sector drug shops frequently dispense parenteral anti-malarials in a rural region of Western Uganda
Source: Malar J. 2018 Aug 22;17:305. doi: 10.1186/s12936-018-2454-7 (PMC6106765; doi:10.1186/s12936-018-2454-7)
Supplement: Supplementary file 2 — Additional file 2. Anti-malarial sales tracking form. [file 12936_2018_2454_MOESM2_ESM.docx]

Additional File 2: Anti-malarial sales tracking form

**Pharmacy Shop Name:**  **Start Date:**  **End Date:**

**Directions**: This study is trying to find out how many anti-malarial drugs are bought in the Kasese District, and we need your help to do that. Every time you sell an antimalarial drug, record the date, patient’s sex/age/village of residence, the name of the drug, how many units of the drug were sold, and how many units of the drug are left in stock. Please track this information for one month (30 days).

| **SECTION 1 of 1: Pharmacy/Drug Shop Information** | | | | | | |
| --- | --- | --- | --- | --- | --- | --- |
| **Date** | **Patient Information** | | | **Drug Information** | | |
|  | **Sex** | **Age** | **Village** | **Drug Name** | **Units Sold** | **Units Left** |
|  | 🞎M 🞎F |  |  | 🞎 Coartem 🞎 Quinine (oral) 🞎 Quinine (IV) 🞎 Quinine (syrup) 🞎 Fansidar 🞎 Artesunate 🞎 Artemether 🞎 Camoquin 🞎 Other(s): |  |  |
|  | 🞎M 🞎F |  |  | 🞎 Coartem 🞎 Quinine (oral) 🞎 Quinine (IV) 🞎 Quinine (syrup) 🞎 Fansidar 🞎 Artesunate 🞎 Artemether 🞎 Camoquin 🞎 Other(s): |  |  |
|  | 🞎M 🞎F |  |  | 🞎 Coartem 🞎 Quinine (oral) 🞎 Quinine (IV) 🞎 Quinine (syrup) 🞎 Fansidar 🞎 Artesunate 🞎 Artemether 🞎 Camoquin 🞎 Other(s): |  |  |
|  | 🞎M 🞎F |  |  | 🞎 Coartem 🞎 Quinine (oral) 🞎 Quinine (IV) 🞎 Quinine (syrup) 🞎 Fansidar 🞎 Artesunate 🞎 Artemether 🞎 Camoquin 🞎 Other(s): |  |  |
|  | 🞎M 🞎F |  |  | 🞎 Coartem 🞎 Quinine (oral) 🞎 Quinine (IV) 🞎 Quinine (syrup) 🞎 Fansidar 🞎 Artesunate 🞎 Artemether 🞎 Camoquin 🞎 Other(s): |  |  |
|  | 🞎M 🞎F |  |  | 🞎 Coartem 🞎 Quinine (oral) 🞎 Quinine (IV) 🞎 Quinine (syrup) 🞎 Fansidar 🞎 Artesunate 🞎 Artemether 🞎 Camoquin 🞎 Other(s): |  |  |
|  | 🞎M 🞎F |  |  | 🞎 Coartem 🞎 Quinine (oral) 🞎 Quinine (IV) 🞎 Quinine (syrup) 🞎 Fansidar 🞎 Artesunate 🞎 Artemether 🞎 Camoquin 🞎 Other(s): |  |  |
|  | 🞎M 🞎F |  |  | 🞎 Coartem 🞎 Quinine (oral) 🞎 Quinine (IV) 🞎 Quinine (syrup) 🞎 Fansidar 🞎 Artesunate 🞎 Artemether 🞎 Camoquin 🞎 Other(s): |  |  |
|  | 🞎M 🞎F |  |  | 🞎 Coartem 🞎 Quinine (oral) 🞎 Quinine (IV) 🞎 Quinine (syrup) 🞎 Fansidar 🞎 Artesunate 🞎 Artemether 🞎 Camoquin 🞎 Other(s): |  |  |
|  | 🞎M 🞎F |  |  | 🞎 Coartem 🞎 Quinine (oral) 🞎 Quinine (IV) 🞎 Quinine (syrup) 🞎 Fansidar 🞎 Artesunate 🞎 Artemether 🞎 Camoquin 🞎 Other(s): |  |  |
|  | 🞎M 🞎F |  |  | 🞎 Coartem 🞎 Quinine (oral) 🞎 Quinine (IV) 🞎 Quinine (syrup) 🞎 Fansidar 🞎 Artesunate 🞎 Artemether 🞎 Camoquin 🞎 Other(s): |  |  |
|  | 🞎M 🞎F |  |  | 🞎 Coartem 🞎 Quinine (oral) 🞎 Quinine (IV) 🞎 Quinine (syrup) 🞎 Fansidar 🞎 Artesunate 🞎 Artemether 🞎 Camoquin 🞎 Other(s): |  |  |
|  | 🞎M 🞎F |  |  | 🞎 Coartem 🞎 Quinine (oral) 🞎 Quinine (IV) 🞎 Quinine (syrup) 🞎 Fansidar 🞎 Artesunate 🞎 Artemether 🞎 Camoquin 🞎 Other(s): |  |  |
|  | 🞎M 🞎F |  |  | 🞎 Coartem 🞎 Quinine (oral) 🞎 Quinine (IV) 🞎 Quinine (syrup) 🞎 Fansidar 🞎 Artesunate 🞎 Artemether 🞎 Camoquin 🞎 Other(s): |  |  |
|  | 🞎M 🞎F |  |  | 🞎 Coartem 🞎 Quinine (oral) 🞎 Quinine (IV) 🞎 Quinine (syrup) 🞎 Fansidar 🞎 Artesunate 🞎 Artemether 🞎 Camoquin 🞎 Other(s): |  |  |
|  | 🞎M 🞎F |  |  | 🞎 Coartem 🞎 Quinine (oral) 🞎 Quinine (IV) 🞎 Quinine (syrup) 🞎 Fansidar 🞎 Artesunate 🞎 Artemether 🞎 Camoquin 🞎 Other(s): |  |  |
|  | 🞎M 🞎F |  |  | 🞎 Coartem 🞎 Quinine (oral) 🞎 Quinine (IV) 🞎 Quinine (syrup) 🞎 Fansidar 🞎 Artesunate 🞎 Artemether 🞎 Camoquin 🞎 Other(s): |  |  |
|  | 🞎M 🞎F |  |  | 🞎 Coartem 🞎 Quinine (oral) 🞎 Quinine (IV) 🞎 Quinine (syrup) 🞎 Fansidar 🞎 Artesunate 🞎 Artemether 🞎 Camoquin 🞎 Other(s): |  |  |
|  | 🞎M 🞎F |  |  | 🞎 Coartem 🞎 Quinine (oral) 🞎 Quinine (IV) 🞎 Quinine (syrup) 🞎 Fansidar 🞎 Artesunate 🞎 Artemether 🞎 Camoquin 🞎 Other(s): |  |  |
|  | 🞎M 🞎F |  |  | 🞎 Coartem 🞎 Quinine (oral) 🞎 Quinine (IV) 🞎 Quinine (syrup) 🞎 Fansidar 🞎 Artesunate 🞎 Artemether 🞎 Camoquin 🞎 Other(s): |  |  |
|  | 🞎M 🞎F |  |  | 🞎 Coartem 🞎 Quinine (oral) 🞎 Quinine (IV) 🞎 Quinine (syrup) 🞎 Fansidar 🞎 Artesunate 🞎 Artemether 🞎 Camoquin 🞎 Other(s): |  |  |
|  | 🞎M 🞎F |  |  | 🞎 Coartem 🞎 Quinine (oral) 🞎 Quinine (IV) 🞎 Quinine (syrup) 🞎 Fansidar 🞎 Artesunate 🞎 Artemether 🞎 Camoquin 🞎 Other(s): |  |  |
|  | 🞎M 🞎F |  |  | 🞎 Coartem 🞎 Quinine (oral) 🞎 Quinine (IV) 🞎 Quinine (syrup) 🞎 Fansidar 🞎 Artesunate 🞎 Artemether 🞎 Camoquin 🞎 Other(s): |  |  |
|  | 🞎M 🞎F |  |  | 🞎 Coartem 🞎 Quinine (oral) 🞎 Quinine (IV) 🞎 Quinine (syrup) 🞎 Fansidar 🞎 Artesunate 🞎 Artemether 🞎 Camoquin 🞎 Other(s): |  |  |
|  | 🞎M 🞎F |  |  | 🞎 Coartem 🞎 Quinine (oral) 🞎 Quinine (IV) 🞎 Quinine (syrup) 🞎 Fansidar 🞎 Artesunate 🞎 Artemether 🞎 Camoquin 🞎 Other(s): |  |  |
|  | 🞎M 🞎F |  |  | 🞎 Coartem 🞎 Quinine (oral) 🞎 Quinine (IV) 🞎 Quinine (syrup) 🞎 Fansidar 🞎 Artesunate 🞎 Artemether 🞎 Camoquin 🞎 Other(s): |  |  |
|  | 🞎M 🞎F |  |  | 🞎 Coartem 🞎 Quinine (oral) 🞎 Quinine (IV) 🞎 Quinine (syrup) 🞎 Fansidar 🞎 Artesunate 🞎 Artemether 🞎 Camoquin 🞎 Other(s): |  |  |
|  | 🞎M 🞎F |  |  | 🞎 Coartem 🞎 Quinine (oral) 🞎 Quinine (IV) 🞎 Quinine (syrup) 🞎 Fansidar 🞎 Artesunate 🞎 Artemether 🞎 Camoquin 🞎 Other(s): |  |  |
|  | 🞎M 🞎F |  |  | 🞎 Coartem 🞎 Quinine (oral) 🞎 Quinine (IV) 🞎 Quinine (syrup) 🞎 Fansidar 🞎 Artesunate 🞎 Artemether 🞎 Camoquin 🞎 Other(s): |  |  |
|  | 🞎M 🞎F |  |  | 🞎 Coartem 🞎 Quinine (oral) 🞎 Quinine (IV) 🞎 Quinine (syrup) 🞎 Fansidar 🞎 Artesunate 🞎 Artemether 🞎 Camoquin 🞎 Other(s): |  |  |
|  | 🞎M 🞎F |  |  | 🞎 Coartem 🞎 Quinine (oral) 🞎 Quinine (IV) 🞎 Quinine (syrup) 🞎 Fansidar 🞎 Artesunate 🞎 Artemether 🞎 Camoquin 🞎 Other(s): |  |  |
|  | 🞎M 🞎F |  |  | 🞎 Coartem 🞎 Quinine (oral) 🞎 Quinine (IV) 🞎 Quinine (syrup) 🞎 Fansidar 🞎 Artesunate 🞎 Artemether 🞎 Camoquin 🞎 Other(s): |  |  |
|  | 🞎M 🞎F |  |  | 🞎 Coartem 🞎 Quinine (oral) 🞎 Quinine (IV) 🞎 Quinine (syrup) 🞎 Fansidar 🞎 Artesunate 🞎 Artemether 🞎 Camoquin 🞎 Other(s): |  |  |
|  | 🞎M 🞎F |  |  | 🞎 Coartem 🞎 Quinine (oral) 🞎 Quinine (IV) 🞎 Quinine (syrup) 🞎 Fansidar 🞎 Artesunate 🞎 Artemether 🞎 Camoquin 🞎 Other(s): |  |  |
|  | 🞎M 🞎F |  |  | 🞎 Coartem 🞎 Quinine (oral) 🞎 Quinine (IV) 🞎 Quinine (syrup) 🞎 Fansidar 🞎 Artesunate 🞎 Artemether 🞎 Camoquin 🞎 Other(s): |  |  |
|  | 🞎M 🞎F |  |  | 🞎 Coartem 🞎 Quinine (oral) 🞎 Quinine (IV) 🞎 Quinine (syrup) 🞎 Fansidar 🞎 Artesunate 🞎 Artemether 🞎 Camoquin 🞎 Other(s): |  |  |
|  | 🞎M 🞎F |  |  | 🞎 Coartem 🞎 Quinine (oral) 🞎 Quinine (IV) 🞎 Quinine (syrup) 🞎 Fansidar 🞎 Artesunate 🞎 Artemether 🞎 Camoquin 🞎 Other(s): |  |  |
|  | 🞎M 🞎F |  |  | 🞎 Coartem 🞎 Quinine (oral) 🞎 Quinine (IV) 🞎 Quinine (syrup) 🞎 Fansidar 🞎 Artesunate 🞎 Artemether 🞎 Camoquin 🞎 Other(s): |  |  |
|  | 🞎M 🞎F |  |  | 🞎 Coartem 🞎 Quinine (oral) 🞎 Quinine (IV) 🞎 Quinine (syrup) 🞎 Fansidar 🞎 Artesunate 🞎 Artemether 🞎 Camoquin 🞎 Other(s): |  |  |
|  | 🞎M 🞎F |  |  | 🞎 Coartem 🞎 Quinine (oral) 🞎 Quinine (IV) 🞎 Quinine (syrup) 🞎 Fansidar 🞎 Artesunate 🞎 Artemether 🞎 Camoquin 🞎 Other(s): |  |  |
|  | 🞎M 🞎F |  |  | 🞎 Coartem 🞎 Quinine (oral) 🞎 Quinine (IV) 🞎 Quinine (syrup) 🞎 Fansidar 🞎 Artesunate 🞎 Artemether 🞎 Camoquin 🞎 Other(s): |  |  |
|  | 🞎M 🞎F |  |  | 🞎 Coartem 🞎 Quinine (oral) 🞎 Quinine (IV) 🞎 Quinine (syrup) 🞎 Fansidar 🞎 Artesunate 🞎 Artemether 🞎 Camoquin 🞎 Other(s): |  |  |
|  | 🞎M 🞎F |  |  | 🞎 Coartem 🞎 Quinine (oral) 🞎 Quinine (IV) 🞎 Quinine (syrup) 🞎 Fansidar 🞎 Artesunate 🞎 Artemether 🞎 Camoquin 🞎 Other(s): |  |  |
|  | 🞎M 🞎F |  |  | 🞎 Coartem 🞎 Quinine (oral) 🞎 Quinine (IV) 🞎 Quinine (syrup) 🞎 Fansidar 🞎 Artesunate 🞎 Artemether 🞎 Camoquin 🞎 Other(s): |  |  |
|  | 🞎M 🞎F |  |  | 🞎 Coartem 🞎 Quinine (oral) 🞎 Quinine (IV) 🞎 Quinine (syrup) 🞎 Fansidar 🞎 Artesunate 🞎 Artemether 🞎 Camoquin 🞎 Other(s): |  |  |
|  | 🞎M 🞎F |  |  | 🞎 Coartem 🞎 Quinine (oral) 🞎 Quinine (IV) 🞎 Quinine (syrup) 🞎 Fansidar 🞎 Artesunate 🞎 Artemether 🞎 Camoquin 🞎 Other(s): |  |  |
|  | 🞎M 🞎F |  |  | 🞎 Coartem 🞎 Quinine (oral) 🞎 Quinine (IV) 🞎 Quinine (syrup) 🞎 Fansidar 🞎 Artesunate 🞎 Artemether 🞎 Camoquin 🞎 Other(s): |  |  |
|  | 🞎M 🞎F |  |  | 🞎 Coartem 🞎 Quinine (oral) 🞎 Quinine (IV) 🞎 Quinine (syrup) 🞎 Fansidar 🞎 Artesunate 🞎 Artemether 🞎 Camoquin 🞎 Other(s): |  |  |
|  | 🞎M 🞎F |  |  | 🞎 Coartem 🞎 Quinine (oral) 🞎 Quinine (IV) 🞎 Quinine (syrup) 🞎 Fansidar 🞎 Artesunate 🞎 Artemether 🞎 Camoquin 🞎 Other(s): |  |  |
|  | 🞎M 🞎F |  |  | 🞎 Coartem 🞎 Quinine (oral) 🞎 Quinine (IV) 🞎 Quinine (syrup) 🞎 Fansidar 🞎 Artesunate 🞎 Artemether 🞎 Camoquin 🞎 Other(s): |  |  |
|  | 🞎M 🞎F |  |  | 🞎 Coartem 🞎 Quinine (oral) 🞎 Quinine (IV) 🞎 Quinine (syrup) 🞎 Fansidar 🞎 Artesunate 🞎 Artemether 🞎 Camoquin 🞎 Other(s): |  |  |
|  | 🞎M 🞎F |  |  | 🞎 Coartem 🞎 Quinine (oral) 🞎 Quinine (IV) 🞎 Quinine (syrup) 🞎 Fansidar 🞎 Artesunate 🞎 Artemether 🞎 Camoquin 🞎 Other(s): |  |  |
|  | 🞎M 🞎F |  |  | 🞎 Coartem 🞎 Quinine (oral) 🞎 Quinine (IV) 🞎 Quinine (syrup) 🞎 Fansidar 🞎 Artesunate 🞎 Artemether 🞎 Camoquin 🞎 Other(s): |  |  |
|  | 🞎M 🞎F |  |  | 🞎 Coartem 🞎 Quinine (oral) 🞎 Quinine (IV) 🞎 Quinine (syrup) 🞎 Fansidar 🞎 Artesunate 🞎 Artemether 🞎 Camoquin 🞎 Other(s): |  |  |
|  | 🞎M 🞎F |  |  | 🞎 Coartem 🞎 Quinine (oral) 🞎 Quinine (IV) 🞎 Quinine (syrup) 🞎 Fansidar 🞎 Artesunate 🞎 Artemether 🞎 Camoquin 🞎 Other(s): |  |  |
|  | 🞎M 🞎F |  |  | 🞎 Coartem 🞎 Quinine (oral) 🞎 Quinine (IV) 🞎 Quinine (syrup) 🞎 Fansidar 🞎 Artesunate 🞎 Artemether 🞎 Camoquin 🞎 Other(s): |  |  |
|  | 🞎M 🞎F |  |  | 🞎 Coartem 🞎 Quinine (oral) 🞎 Quinine (IV) 🞎 Quinine (syrup) 🞎 Fansidar 🞎 Artesunate 🞎 Artemether 🞎 Camoquin 🞎 Other(s): |  |  |
|  | 🞎M 🞎F |  |  | 🞎 Coartem 🞎 Quinine (oral) 🞎 Quinine (IV) 🞎 Quinine (syrup) 🞎 Fansidar 🞎 Artesunate 🞎 Artemether 🞎 Camoquin 🞎 Other(s): |  |  |
|  | 🞎M 🞎F |  |  | 🞎 Coartem 🞎 Quinine (oral) 🞎 Quinine (IV) 🞎 Quinine (syrup) 🞎 Fansidar 🞎 Artesunate 🞎 Artemether 🞎 Camoquin 🞎 Other(s): |  |  |
|  | 🞎M 🞎F |  |  | 🞎 Coartem 🞎 Quinine (oral) 🞎 Quinine (IV) 🞎 Quinine (syrup) 🞎 Fansidar 🞎 Artesunate 🞎 Artemether 🞎 Camoquin 🞎 Other(s): |  |  |
